# Increasing aggressiveness of patient-derived xenograft models of cervix carcinoma during serial transplantation

**DOI:** 10.18632/oncotarget.24783

**Published:** 2018-04-20

**Authors:** Catherine S. Wegner, Anette Hauge, Lise Mari K. Andersen, Ruixia Huang, Trude G. Simonsen, Jon-Vidar Gaustad, Einar K. Rofstad

**Affiliations:** ^1^ Group of Radiation Biology and Tumor Physiology, Department of Radiation Biology, Institute for Cancer Research, Oslo University Hospital, Oslo, Norway

**Keywords:** cervix cancer, angiogenesis, cancer stem cells, hypoxia, metastasis

## Abstract

Four patient-derived xenograft (PDX) models (BK-12, ED-15, HL-16, LA-19) of carcinoma of the uterine cervix have been developed in our laboratory, and their stability during serial transplantation *in vivo* was investigated in this study. Two frozen cell stocks were established, one from xenografted tumors in passage 2 (early generation) and the other from xenografted tumors transplanted serially in mice for approximately two years (late generation), and the biology of late generation tumors was compared with that of early generation tumors. Late generation tumors showed higher incidence of lymph node metastases than early generation tumors in three models (ED-15, HL-16, LA-19), and the increased metastatic propensity was associated with increased tumor growth rate, increased microvascular density, and increased expression of angiogenesis-related and cancer stem cell-related genes. Furthermore, late generation tumors showed decreased fraction of pimonidazole-positive tissue (*i.e.*, decreased fraction of hypoxic tissue) in two models (HL-16, LA-19) and decreased fraction of collagen-I-positive tissue (i.e., less extensive extracellular matrix) in two models (ED-15, HL-16). This study showed that serially transplanted PDXs may not necessarily mirror the donor patients’ diseases, and consequently, proper use of serially transplanted PDX models in translational cancer research requires careful molecular monitoring of the models.

## INTRODUCTION

Preclinical xenograft models of human cancer reflecting the biology of the donor patients’ tumors are essential tools for conducting clinically relevant cancer research. Xenografted tumors initiated from well characterized established cell lines are used frequently to study molecular pathways of cancer evolution as well as antitumor effects of therapeutic agents. However, it has been revealed that cell line-derived xenograft (CDX) models do not mirror accurately the biology of human tumors, and furthermore, the response to treatment of CDX models may fail to predict the treatment response in cancer patients [1, 2].

The limited clinical relevance of CDX models has initiated an increased interest in establishing improved cancer models by transplanting surgical specimens from human tumors directly into immune-deficient mice [3, 4]. These models are referred to as patient-derived xenograft (PDX) models and are maintained *in vivo* without being exposed to cell culture conditions *in vitro*. It has been shown that PDX models retain the histopathological and genotypic characteristics of the donor patients’ tumor tissue, and comparative studies including several tumor types have revealed that PDX models and donor patients’ tumors show similar responses to treatment [3–6]. Consequently, it has been suggested that PDXs may be useful cancer models in many disciplines of oncologic research, including identification of novel biomarkers, evaluation of potentially useful anticancer agents, and the development of strategies for precision cancer medicine [1, 3, 4, 7, 8].

In many laboratories, PDX models of human cancer are maintained by serial transplantation in immune-deficient mice. It has been reported that PDX models show an unchanged gene expression profile during serial transplantation, and the response to treatment has been seen to be stable over multiple transplantations [9, 10]. However, shortly after Rygaard and Povlsen established the first PDX model of cancer in the congenitally athymic nude mouse in 1969 [11], they and several other investigators observed that the growth rate of human tumor xenografts initiated from surgical specimens increased gradually during serial transplantation *in vivo*, and furthermore, serially transplanted xenografted tumors were shown to develop a stroma consisting of cellular and extracellular matrix components of murine origin [12, 13]. Despite these early observations, little concern has been devoted to the possibility that PDX models of cancer may change significantly in biological properties and treatment response during serial transplantation and, hence, may evolve to a state where they have limited value as clinically relevant tumor models.

Four PDX models of squamous cell carcinoma of the uterine cervix were recently established in our laboratory [14], and it was shown that early generation tumors of these models mirror the histological appearance, angiogenic activity, and metastatic propensity of the donor patients’ tumors [15]. We have observed that the tumor take rate (transplantation efficiency) and the tumor growth rate of these models increase during serial transplantation *in vivo*, and therefore, quantitative studies of possible biological changes induced during serial transplantation were conducted. Two frozen stocks of the PDX models were established, one from xenografted tumors in passage 2 (early generation) and one from xenografted tumors transplanted serially in mice for two years (late generation). In this communication, biological properties of late generation tumors are compared with those of early generation tumors. Several significant differences were observed, and the implications of these findings for the use of PDX models in preclinical cancer research are discussed.

## RESULTS

### Tumor histology did not change during serial transplantation

**Figure 1:**
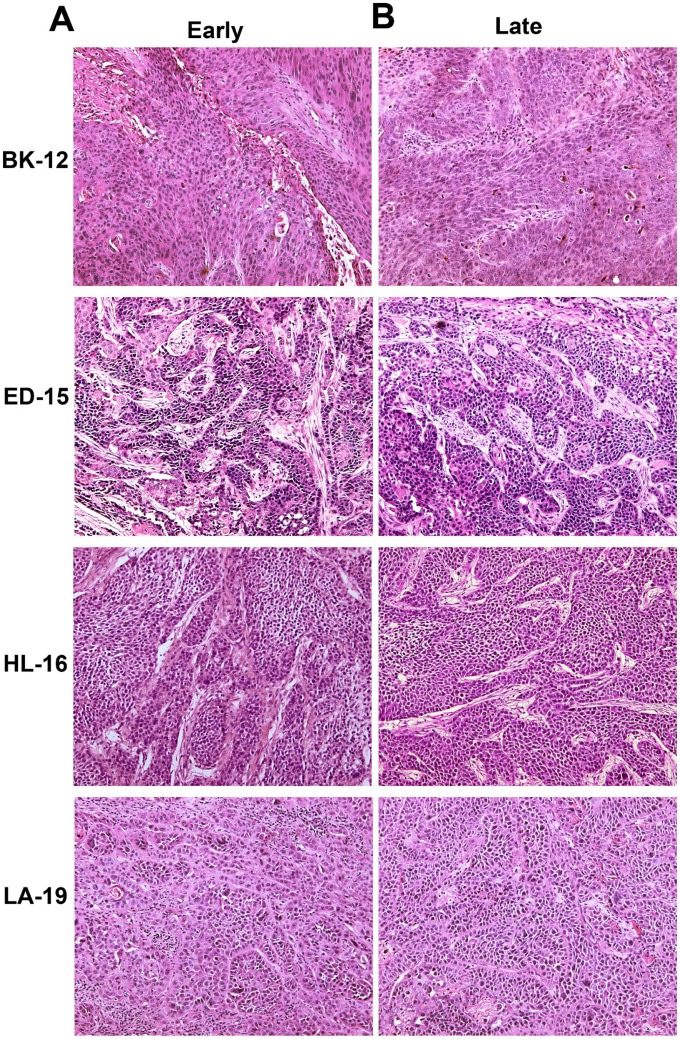
Tumor histology Histological preparations of early (**A**) and late (**B**) generation BK-12, ED-15, HL-16, and LA-19 tumors stained with hematoxylin and eosin.

To investigate whether late generation tumors differed from early generation tumors in histological appearance, histological preparations were stained with hematoxylin and eosin (HE) or immunostained for blood vessels, hypoxic tissue, or collagen-I. HE stained preparations showed that BK-12, ED-15, and LA-19 tumors were moderately differentiated and HL-16 tumors were poorly differentiated, similar to the donor patients’ tumors, and furthermore, late generation tumors were indistinguishable from early generation tumors (Figure 1).

**Figure 2:**
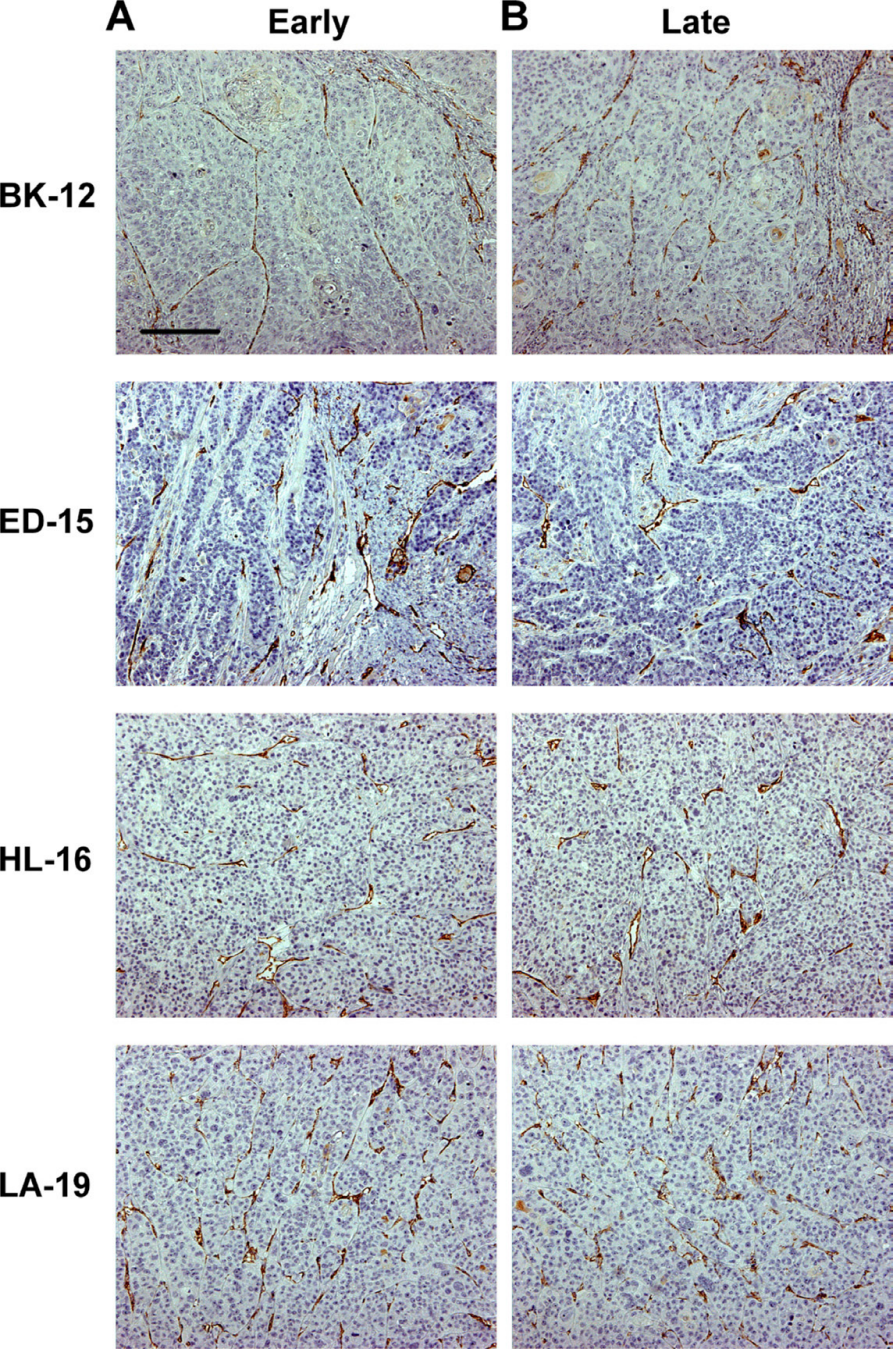
Tumor microvasculature Histological preparations of early (**A**) and late (**B**) generation BK-12, ED-15, HL-16, and LA-19 tumors immunostained for CD31 to visualize blood vessels. Scale bar: 200 μm.

CD31 was used as a marker for blood vessel endothelial cells, and immunohistochemical preparations stained for CD31 revealed that the microvasculature differed among the tumor models. The vessels in BK-12, ED-15, and HL-16 tumors were located primarily within stromal connective tissue, whereas LA-19 tumors frequently showed vessels also in the parenchyma. Moreover, the tumor models differed clearly in staining pattern, suggesting that they developed microvascular networks having significant architectural differences. Differences between early and late generation tumors could not be detected in any of the models (Figure 2), suggesting that the microvasculature did not change significantly during serial transplantation.

**Figure 3:**
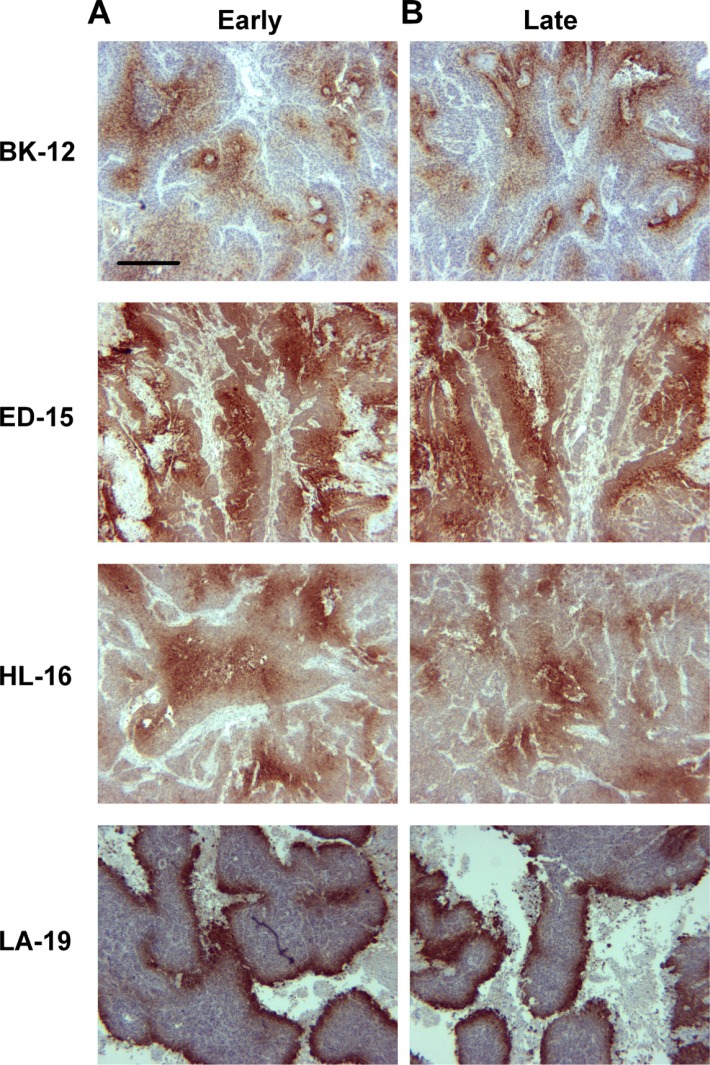
Tumor hypoxia Histological preparations of early (**A**) and late (**B**) generation BK-12, ED-15, HL-16, and LA-19 tumors immunostained for pimonidazole to visualize hypoxic tissue. Scale bar: 400 μm.

Pimonidazole was used as a hypoxia marker, and immunohistochemical preparations stained for pimonidazole revealed that the staining pattern differed among the tumor models. BK-12 and ED-15 tumors were characterized by perinecrotic as well as focal staining, HL-16 tumors showed a predominant focal staining pattern, and LA-19 tumors developed large regions with necrotic tissue and showed primarily perinecrotic staining. Late generation tumors did not differ from early generation tumors in staining pattern (Figure 3), suggesting that serial transplantation did not induce significant changes in tumor oxygen distribution.

**Figure 4:**
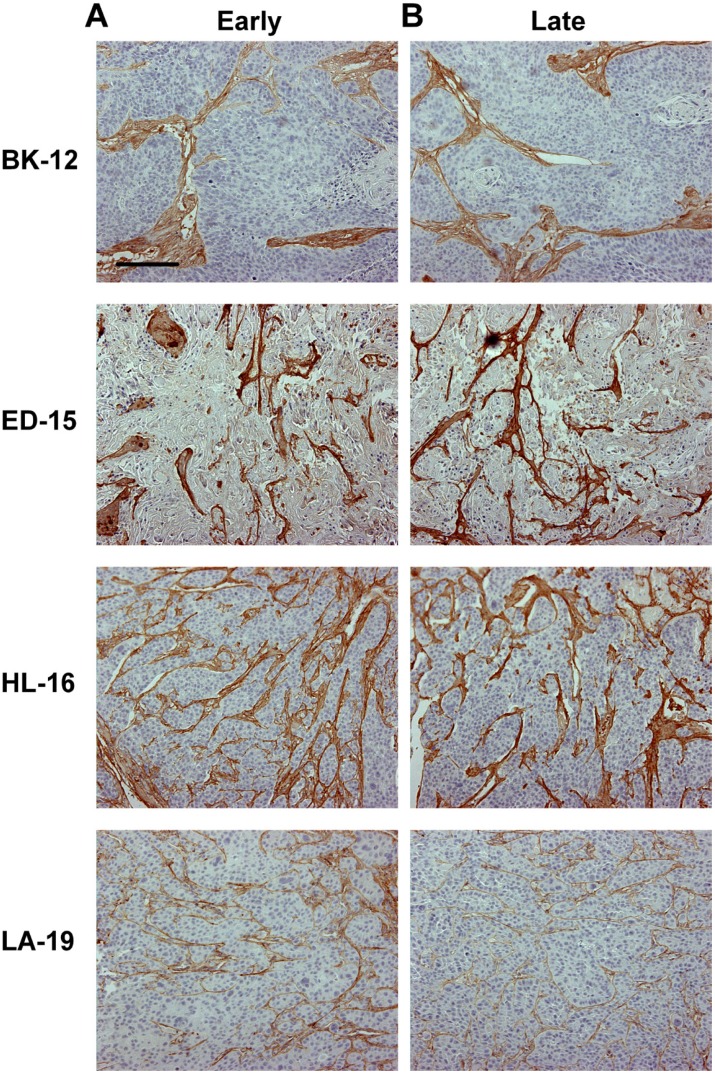
Tumor extracellular matrix Histological preparations of early (**A**) and late (**B**) generation BK-12, ED-15, HL-16, and LA-19 tumors immunostained for collagen-I to visualize the extracellular matrix. Scale bar: 200 μm.

Collagen-I has been identified as the most prominent component of the extracellular matrix of the tumor models [14], and to investigate whether the extracellular matrix changed during serial transplantation, histological sections of early and late generation tumors were immunostained for collagen-I. The extracellular matrix differed substantially among the models; BK-12 tumors showed particularly thick filament bundles at low density, whereas the filament bundles in LA-19 tumors were thinner and more numerous. The staining pattern did not differ between early and late generation tumors in any of the models (Figure 4), suggesting that the basic structure of the extracellular matrix was retained during serial transplantation.

### Late generation tumors showed increased growth rate, angiogenesis, and metastasis

**Figure 5:**
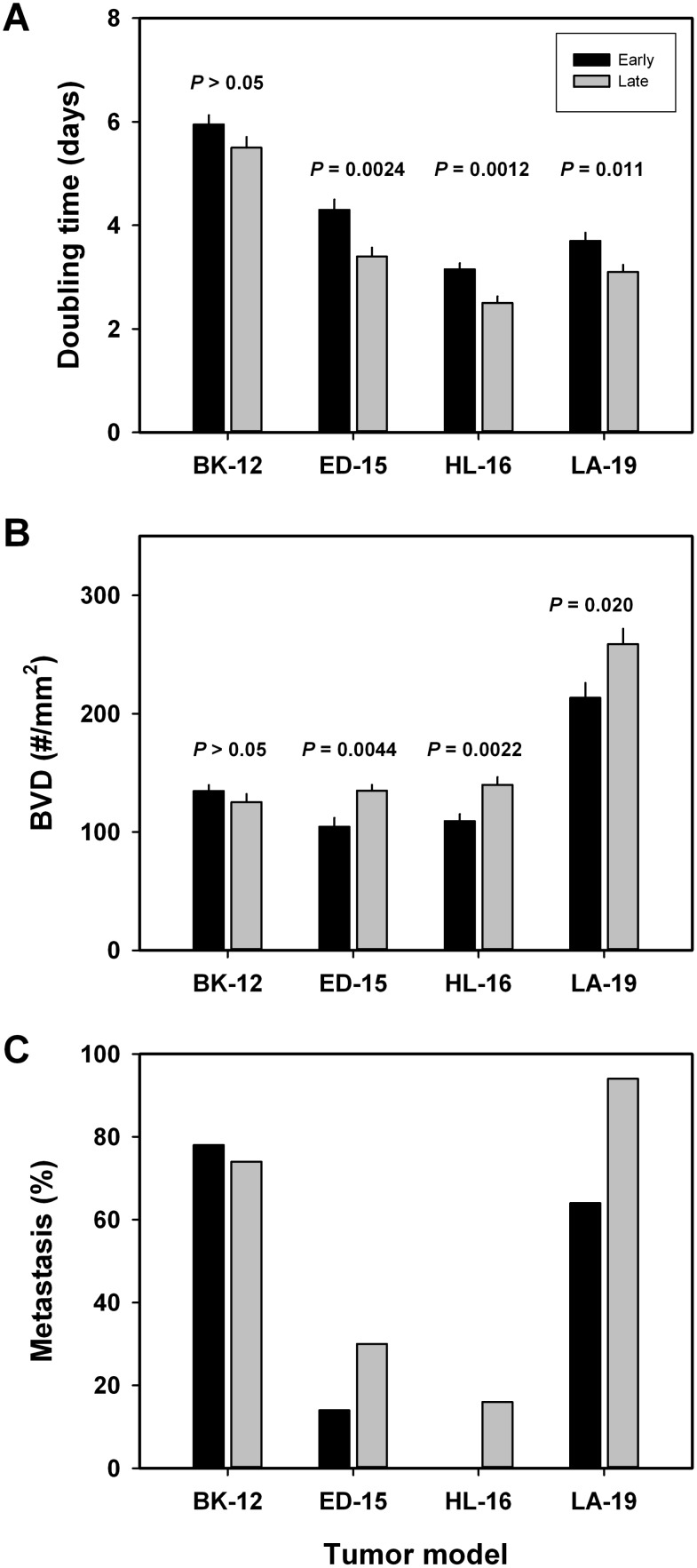
Tumor aggressiveness Volume doubling time (**A**), blood vessel density (**B**), and metastatic frequency (**C**) of early and late generation BK-12, ED-15, HL-16, and LA-19 tumors. Columns and bars in (A and B): mean ± SE (*n* = 20). Columns and bars in (C) percentage of mice with at least one lymph node metastasis (*n* = 55–70).

Measurements of tumor growth revealed that the growth rate tended to increase during serial transplantation. Thus, the volume doubling time was significantly shorter in late than in early generation tumors of the ED-15 (*P* = 0.0024), HL-16 (*P* = 0.0012), and LA-19 (*P* = 0.011) models, whereas a significant difference was not seen in the BK-12 model (Figure 5A). The increase in growth rate was associated with increased angiogenesis. Late generation tumors showed significantly higher blood vessel density than early generation tumors, both in the ED-15 (*P* = 0.0044), HL-16 (*P* = 0.0022), and LA-19 (*P* = 0.020) models, but not in the BK-12 model (Figure 5B). Furthermore, the tumor models that showed increased tumor growth rate and angiogenesis after serial transplantation also showed increased metastatic propensity (Figure 5C), primarily to the medial iliac lymph nodes and the renal lymph nodes. The incidence of lymph node metastasis (the percentage of mice that showed metastatic growth) had increased by a factor of ~2.1 in the ED-15 model and by a factor of ~1.5 in the LA-19 model. Approximately 16% of the late generation HL-16 tumors developed lymph node metastases, whereas early generation HL-16 tumors did not metastasize. In the BK-12 model, the incidence of lymph node metastasis was similar in late and early generation tumors.

### Late generation tumors showed decreased fractions of pimonidazole-positive tissue and collagen-I-positive tissue

**Figure 6:**
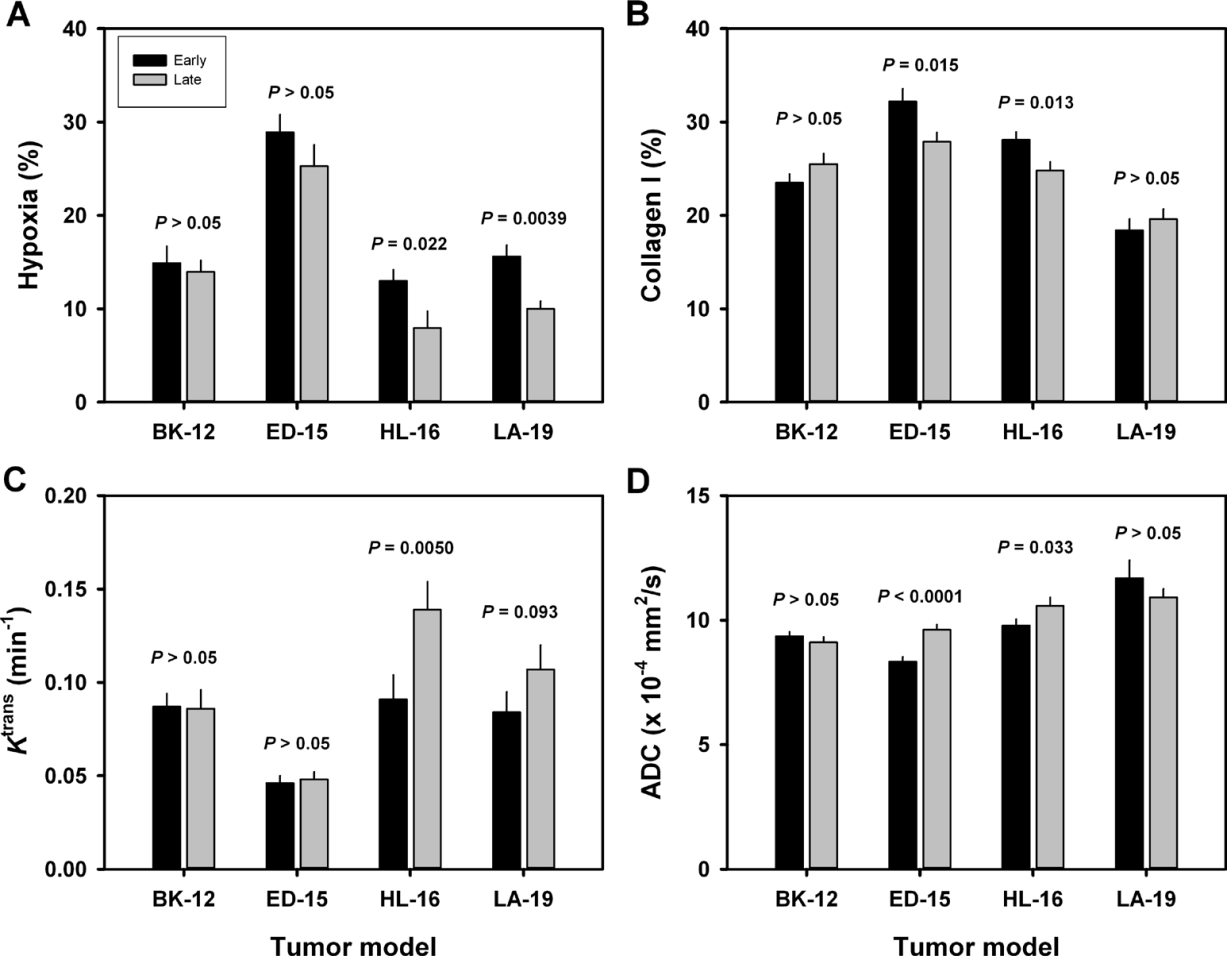
Tumor microenvironment Fraction of hypoxic (pimonidazole-positive) tissue (**A**), fraction of collagen-I-positive tissue (**B**), *K*^trans^ (volume transfer constant of Gd-DOTA) (**C**), and ADC (apparent diffusion coefficient) (**D**) of early and late generation BK-12, ED-15, HL-16, and LA-19 tumors. Columns and bars: mean ± SE (*n* = 12–28).

To investigate whether the extent of tumor hypoxia and the magnitude of the extracellular matrix changed during serial transplantation, immunohistochemical preparations stained for pimonidazole or collagen-I were subjected to quantitative studies. These studies revealed that the fraction of pimonidazole-positive tissue was lower in late than in early generation tumors of the HL-16 (*P* = 0.022) and LA-19 (*P* = 0.0039) models, whereas late and early generation tumors of the BK-12 and ED-15 models did not differ significantly in hypoxic fraction (Figure 6A). Moreover, the fraction of collagen-I-positive tissue was lower in late than in early generation tumors of the ED-15 (*P* = 0.015) and HL-16 (*P* = 0.013) models, but did not differ significantly between late and early generation tumors of the BK-12 and LA-19 models (Figure 6B).

**Figure 7:**
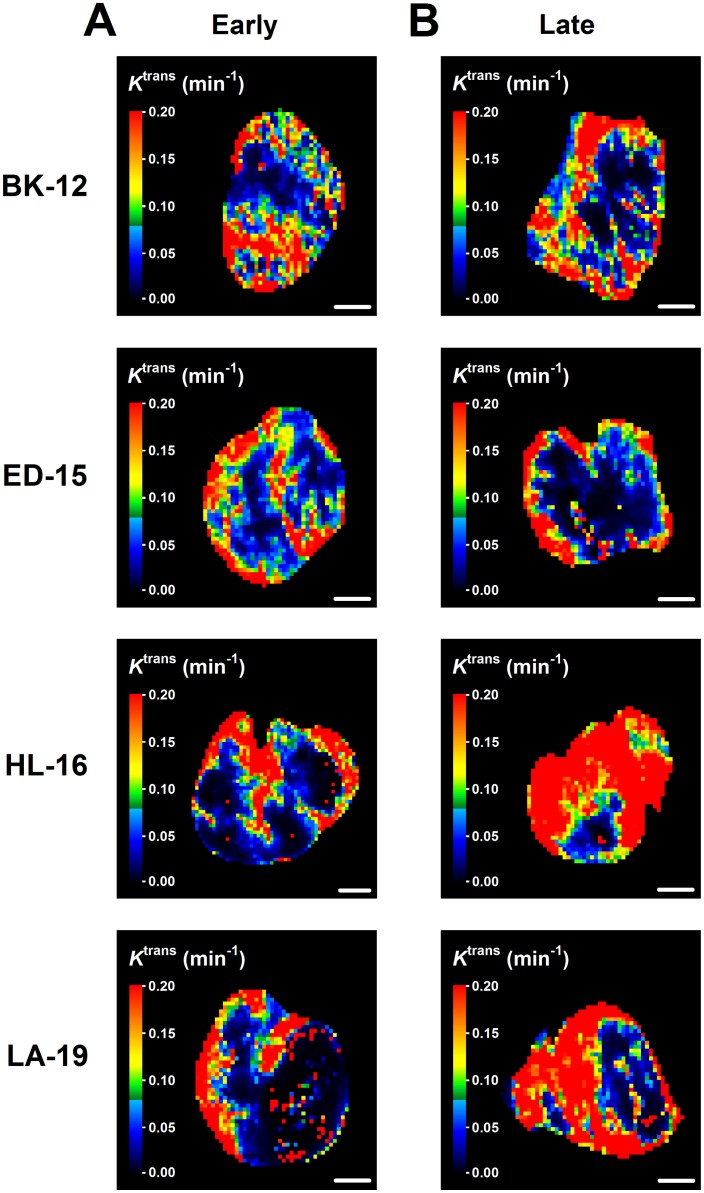
Dynamic contrast-enhanced magnetic resonance imaging-derived parametric images *K*^trans^ (volume transfer constant of Gd-DOTA) images of early (**A**) and late (**B**) generation BK-12, ED-15, HL-16, and LA-19 tumors. Scale bar: 2.0 mm.

**Figure 8:**
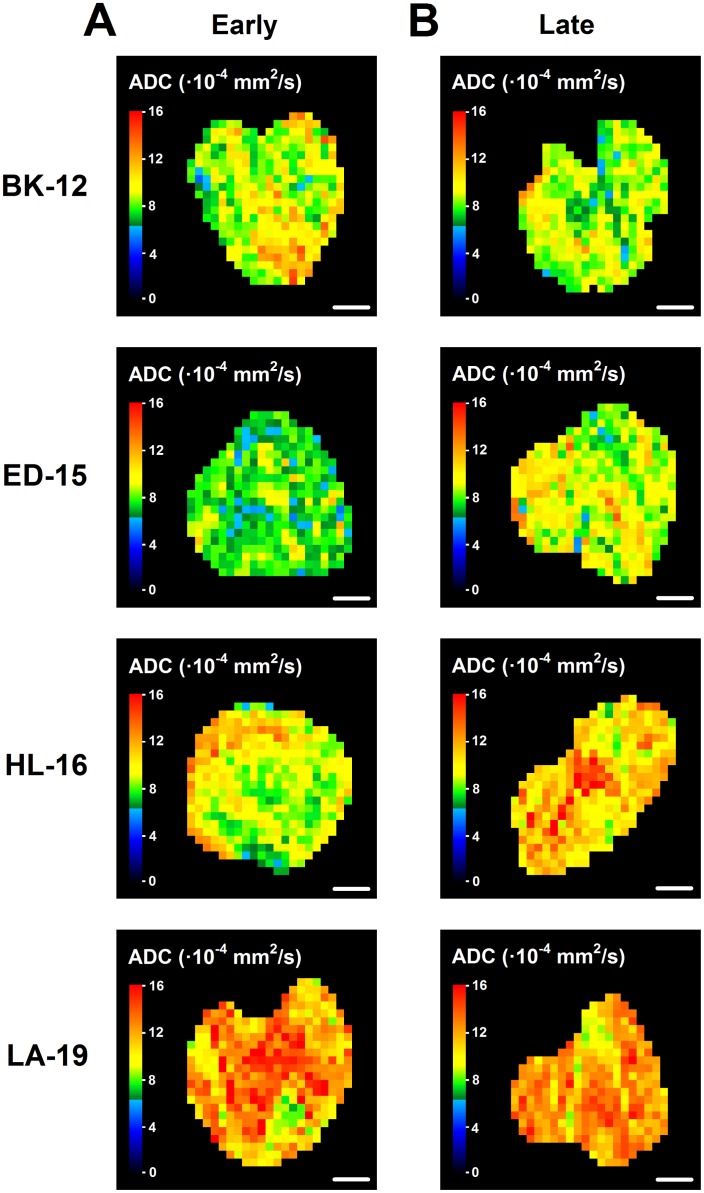
Diffusion-weighted magnetic resonance imaging-derived parametric images ADC (apparent diffusion coefficient) images of early (**A**) and late (**B**) generation BK-12, ED-15, HL-16, and LA-19 tumors. Scale bar: 2.0 mm.

Dynamic contrast-enhanced magnetic resonance imaging (DCE-MRI) and diffusion-weighted magnetic resonance imaging (DW-MRI) of CDXs of cervix carcinoma have been conducted in our laboratory, and these studies showed that *K*^trans^ (the volume transfer constant of Gd-DOTA) was inversely correlated to fraction of hypoxic tissue [16], and ADC (the apparent diffusion coefficient) was inversely correlated to fraction of collagen-I-positive tissue [17]. We hypothesized that similar correlations existed for the PDX models, and therefore, serial transplantation-induced changes in the extent of tumor hypoxia and the magnitude of the extracellular matrix were examined further by subjecting early and late generation tumors to DCE-MRI and DW-MRI. In accordance with our hypothesis, *K*^trans^ was significantly higher in late than in early generation tumors of the HL-16 model (*P* = 0.0050) and on the borderline of being significantly higher in late than in early generation tumors of the LA-19 model (*P* = 0.093), but did not differ significantly between late and early generation tumors of the BK-12 and ED-16 models (Figure 6C). Moreover, ADC was higher in late than in early generation tumors of the ED-15 (*P* < 0.0001) and HL-16 (*P* = 0.033) models, whereas late and early generation tumors of the BK-12 and LA-19 models did not differ significantly in ADC (Figure 6D). Consequently, the immunohistochemical and MRI data were in accordance, providing strong evidence that the hypoxic and extracellular matrix compartments of the tumors changed during serial transplantation *in vivo*. Representative examples of *K*^trans^ and ADC images of early and late generation tumors are presented in Figure 7 and Figure 8, respectively.

### Late generation tumors showed increased gene expression

**Figure 9:**
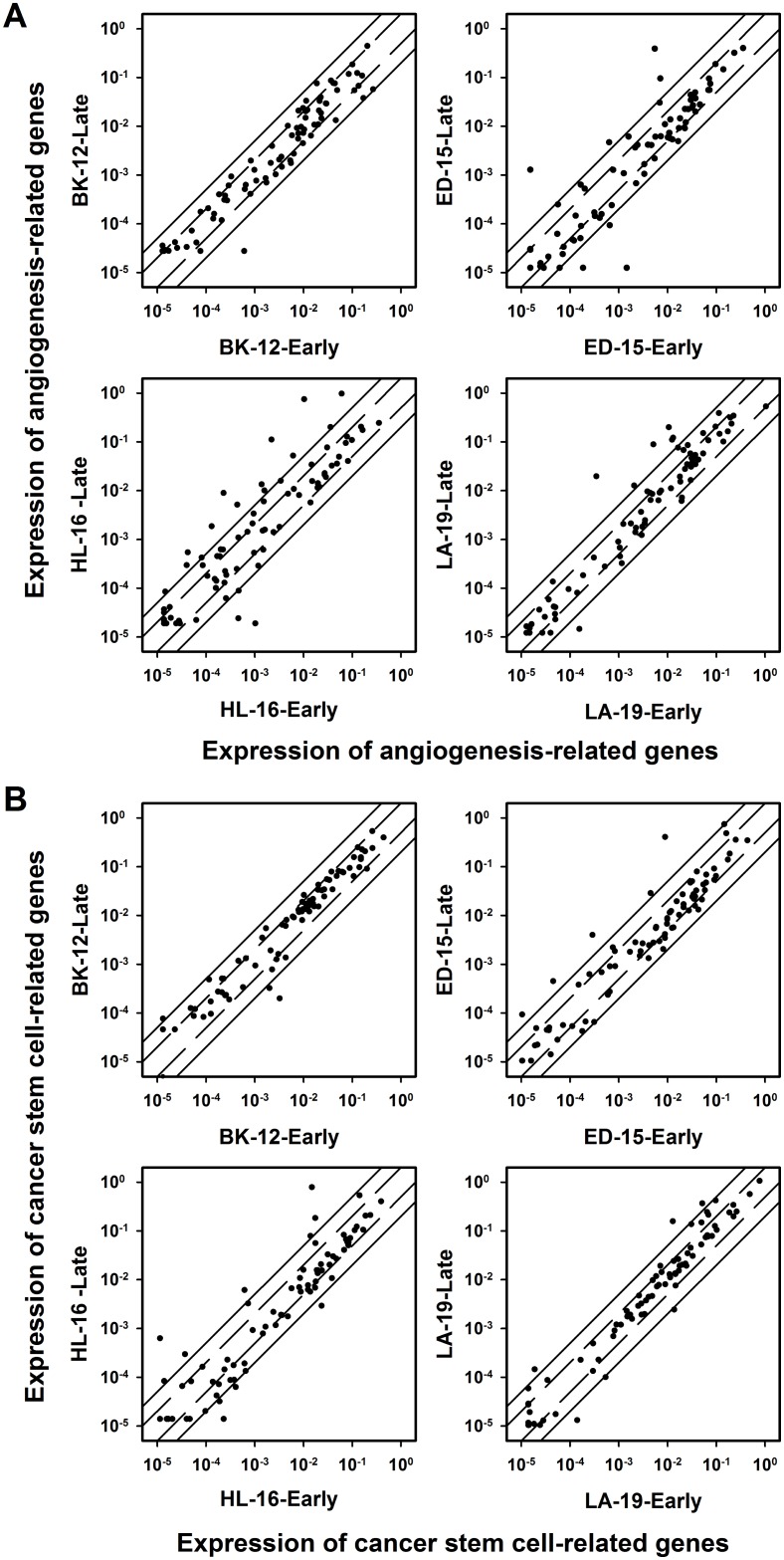
Gene expression The expression of angiogenesis-related genes (**A**) and cancer stem cell-related genes (**B**) in late generation *versus* early generation BK-12, ED-15, HL-16, and LA-19 tumors. Symbols: relative expression of single genes. Solid lines: 5-fold difference in expression level between late and early generation tumors. Dashed lines: 2-fold difference in expression level between late and early generation tumors. The expression levels were generally higher in late generation than in early generation tumors of the ED-15, HL-16, and LA-19 models, whereas a similar difference could not be detected for BK-12 tumors.

**Figure 10:**
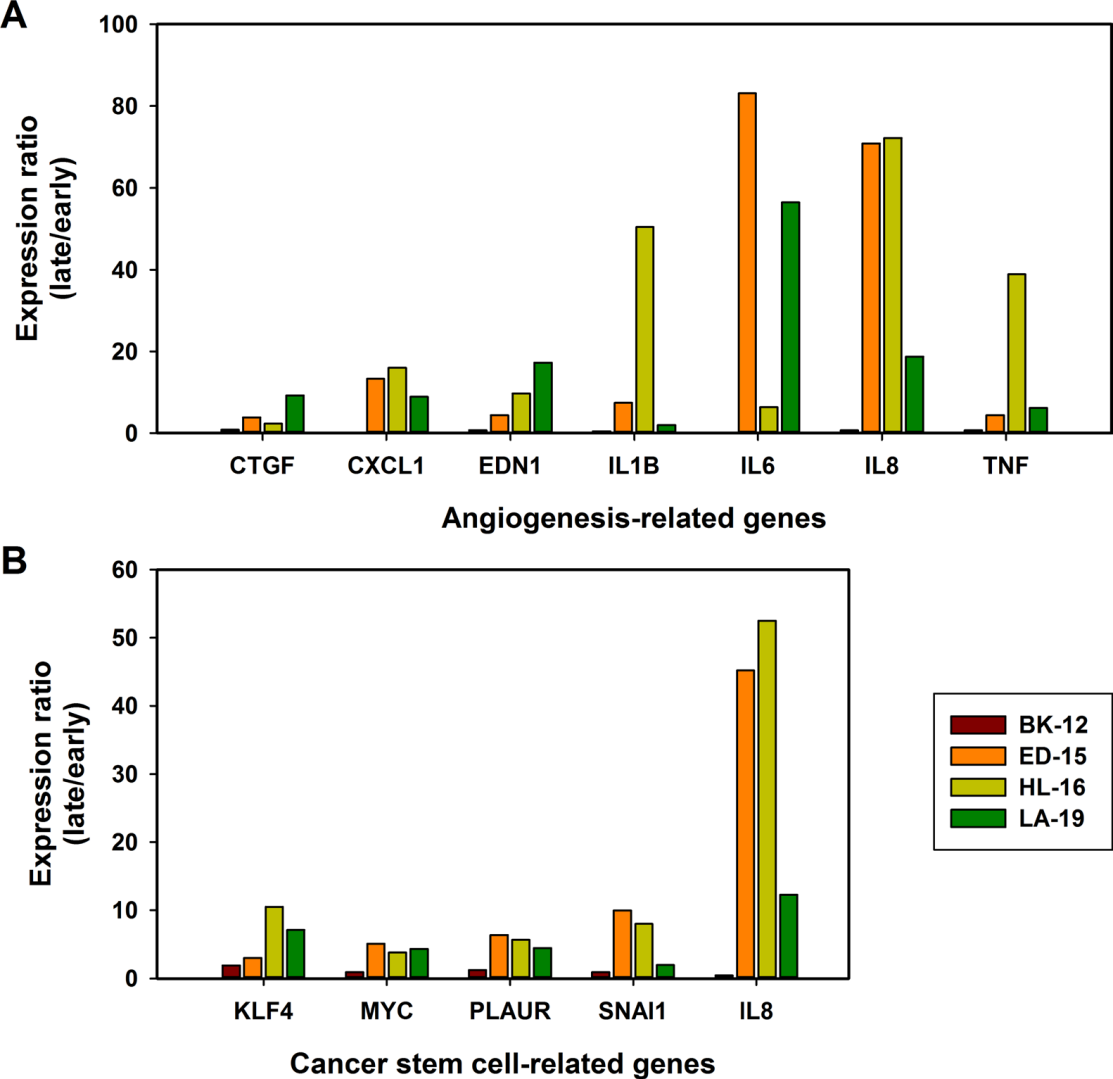
Changes in gene expression The gene expression ratio of late to early generation tumors of seven angiogenesis-related genes (**A**) and five cancer stem cell-related genes (**B**). The genes were significantly up-regulated in late generation tumors of the ED-15, HL-16, and LA-19 models, but not in late generation tumors of the BK-12 model. Columns: mean of three late generation tumors divided by mean of three early generation tumors.

To investigate whether the changes in tumor growth, angiogenesis, metastasis, hypoxia, and extracellular matrix induced during serial transplantation reflected changes in transcriptional regulation of angiogenesis-related and/or cancer stem cell-related genes, early and late generation tumors were subjected to quantitative PCR using commercially available 84-gene arrays. In the BK-12 model, the expression levels were similar for late and early generation tumors, whereas late generation tumors generally showed higher expression than early generation tumors in the ED-15, HL-16, and LA-19 models, both for angiogenesis-related genes (Figure 9A) and cancer stem cell-related genes (Figure 9B). The ED-15, HL-16, and LA-19 models had seven angiogenesis-related genes in common and five cancer stem cell-related genes in common that were significantly up-regulated in late generation tumors (Table 1). The expression ratios of late to early generation tumors of these genes differed substantially among the tumor models and the genes, irrespective of whether the angiogenesis-related genes (Figure 10A) or the cancer stem cell-related genes (Figure 10B) were considered.

**Table 1:** Genes showing significantly higher expression in late than in early generation tumors of the ED-15, HL-16, and LA-19 cervix cancer models

*Angiogenesis-related genes*
CTGF (Connective tissue growth factor)
CXCL1 (Chemokine (C-X-C motif) ligand 1)
EDN1 (Endothelin 1)
IL1B (Interleukin 1 beta)
IL6 (Interleukin 6)
IL8 (Interleukin 8)
TNF (Tumor necrosis factor)
*Stem cell-related genes*
KLF4 (Kruppel like factor 4)
MYC (MYC proto-oncogene, bHLH transcription factor)
PLAUR (Plasminogen activator, urokinase receptor)
SNAI1 (Snail family transcriptional repressor 1)
IL8 (Interleukin 8)

## DISCUSSION

PDX models of cancer have been reported to show phenotypic characteristics and genetic and molecular expression profiles similar to tumors in patients, whereas CDX models do not, and consequently, PDX models are increasingly being used to study tumor biology and response to treatment [1–8]. Most studies are conducted with PDX models of colorectal, melanoma, breast, lung, and pancreatic tumors, primarily because a large number of PDX models are available for these cancer types. More recently, PDX models have also been established for other types of cancer, including carcinoma of the uterine cervix [14, 18, 19]. Similar to most PDX models, the PDX models of cervix cancer demonstrate a histological appearance with a cellular complexity and stromal architecture that mirrors their human counterparts.

Despite the fact that PDXs show increasing growth rate and develop a murine stroma during serial transplantation [12, 13], it has been claimed that PDX models transplanted serially for many generations may preserve the histological appearance, gene expression profile, and treatment response of the donor patients’ tumors [9, 10, 20]. Our study revealed that although the tumor histology was unchanged after two years of serial transplantation, the tumors of the ED-15, HL-16, and LA-19 models had developed a more aggressive phenotype. The late generation tumors showed increased growth rate, increased blood vessel density, increased metastatic propensity, and increased expression of several angiogenesis-related and cancer stem cell-related genes. Moreover, the fraction of hypoxic tissue and the magnitude of the extracellular matrix were reduced in two models.

The BK-12 model was the only model that did not show significantly changed biological properties after two years of serial transplantation. However, this observation does not imply that serial transplantation has no influence on the biology of the tumors of this model. In a previous investigation, we observed that BK-12 tumors transplanted serially for 15–20 generations (*i.e.*, for more than three years) showed increased growth rate, increased interstitial fluid pressure, decreased lymph vessel density, and altered expression of several genes associated with lymphangiogenesis [15]. BK-12 tumors have longer volume doubling times than ED-15, HL-16, and LA-19 tumors, and because of the low growth rate, BK-12 tumors may require a long period of serial transplantation before significant biological changes can be detected.

It has been recommended that PDXs should be transplanted to orthotopic sites to allow the tumor cells to interact with the most relevant organ microenvironment [21]. In this study, the tumors were transplanted to an intramuscular site rather than to an orthotopic site, primarily because orthotopic transplantation of cervix carcinomas is technically challenging and not well suited for large scale investigations. It is not likely that the biological changes induced during the serial transplantation of ED-15, HL-16, and LA-19 tumors can be attributed to the transplantation site since it has been revealed that serial transplantation can induce changes in the biology of PDXs implanted in other ectopic sites as well as in orthotopic sites. Thus, it has been reported that PDX models of squamous cell carcinoma of the head and neck showed significantly increased tumor growth rate after serial subcutaneous transplantation [22]. Moreover, a study of PDX models of cervix carcinoma revealed that tumor blood vessel density and the incidence of lymph node metastases increased during serial orthotopic transplantation in SCID and NOD/SCID mice [19].

Human tumors transplanted to immune-deficient mice interact with a microenvironment that differs from their original microenvironment, independent of the transplantation site. First, the growth and metastasis of human tumors may be suppressed by immune reactions by the host, and these immune reactions are significantly changed in immune-deficient mice. Second, human tumors develop a stroma consisting of a variety of components, including an extracellular matrix, cancer-associated fibroblasts, immune cells, and blood vessels lined by endothelial cells and a basement membrane, and this stroma is of murine origin in xenografted tumors. Human tumors transplanted serially in immune-deficient mice may thus change their biological properties because the tumor cells gradually adapt to and interact with a new microenvironment. The changes in biological properties of serially transplanted PDXs observed in this study were most likely a consequence of the tumor tissue being exposed to the general microenvironment of immune-deficient mice rather than to the organ-specific microenvironment of muscle tissue.

There is strong evidence that the aggressiveness of tumors is associated with characteristic features of the tumor microenvironment [23]. Studies of a large number of cancer types have revealed that high metastatic propensity and poor outcome of treatment are associated with an extensive, collagen-rich extracellular matrix [24, 25], high fraction of hypoxic tissue [26, 27], or highly elevated blood vessel density [28, 29]. ED-15, HL-16, and LA-19 tumors showed increased growth rate and increased incidence of lymph node metastases after serial transplantation, and this increase was associated with increased blood vessel density. A similar increase in the fraction of collagen-I-positive tissue or the fraction of pimonidazole-positive tissue was not detected, suggesting that the increased aggressiveness was associated with increased angiogenesis rather than elevated collagen-I expression or increased hypoxia.

Moreover, quantitative PCR showed that seven angiogenesis-related genes were significantly up-regulated in late generation ED-15, HL-16, and LA-19 tumors. Four of the genes code for the inflammatory cytokines IL-1B, IL-6, IL-8, and TNF. Inflammatory cytokines promote tumor angiogenesis directly, but also indirectly by inducing increased expression of hypoxia-inducible factor-1 [30]. Elevated expression of inflammatory cytokines is associated with decreased progression-free survival in squamous cell carcinoma of the head and neck [31]. IL-8 plays an essential role in lymph node metastasis of early stage cervix cancer [32], and blocking of IL-8 can abrogate tumor growth and metastasis in CDX models of cervix carcinoma [33]. TNF promotes tumor angiogenesis by up-regulating the expression of several members of the vascular endothelial growth factor family [34]. The CXCL1 chemokine, also highly up-regulated in late generation ED-15, HL-16, and LA-19 tumors, has the ELR motif proximal to the CXC sequence, and all ELR containing CXC chemokines are potent promoters of tumor angiogenesis [35].

Late generation ED-15, HL-16, and LA-19 tumors also showed significantly increased expression of five cancer stem cell-related genes, suggesting that their increased aggressiveness could be caused by an increased fraction of cancer stem cells. Cancer stem cells exhibit an aggressive phenotype [36], and in addition to being inherently highly metastatic, they may facilitate metastasis by promoting angiogenesis and lymphangiogenesis [37, 38]. Three of the up-regulated cancer stem cell-related genes (KLF4, MYC, and SNAI1) are transcription factors involved in rearrangement of the extracellular matrix and in epithelial to mesenchymal transition (EMT), processes that are important in tumor angiogenesis, invasion, and metastasis [39, 40]. It has been suggested that KLF4 together with SNAI1 may promote metastasis by inducing transdifferentiation of tumor cells into endothelial cells by an EMT-dependent mechanism [41, 42], while other studies have suggested that KLF4 also can promote metastasis by EMT-independent mechanisms [43]. IL-8 (the angiogenesis factor discussed above) and PLAUR (also known as uPAR) were the other two genes on the cancer stem cell PCR array that were significantly up-regulated in late generation ED-15, HL-16, and LA-19 tumors. Hypoxia-induced up-regulation of PLAUR has been shown to result in increased lymph node metastasis through degradation of the extracellular matrix in a CDX model of malignant melanoma [44], and the ligand of this receptor (uPA) has been revealed to be a possible biomarker for lymph node metastasis in cervix cancer [45].

The study reported here has significant implications for the use of PDX models in translational cancer research. It shows that PDX models of cancer may acquire a more aggressive phenotype during serial transplantation *in vivo*, and consequently, serially transplanted PDXs may not necessarily mirror the biology and treatment response of the donor patients’ tumors. If serial transplantation cannot be avoided, proper use of PDX models in cancer research requires careful phenotypic and molecular monitoring of the serially transplanted tumor tissue.

## MATERIALS AND METHODS

### Tumor models

Adult (8–12 weeks of age) female BALB/c *nu*/*nu* mice were used as host animals for xenografted tumors. Four PDX models (BK-12, ED-15, HL-16, and LA-19) of squamous cell carcinoma of the uterine cervix, established from patients with FIGO stage IIB disease prior to treatment, were included in the study [14]. These models have been maintained solely *in vivo* by transplanting tumor tissue from the donor patients directly into mice without going by short-term *in vitro* culture, and after the initial transplantation, by serial transplantation of tumor cell aliquots in mice. Two frozen cell stocks of these models have been established, one from xenografted tumors in passage 2 (early generation) and the other from xenografted tumors transplanted serially in mice for approximately two years (late generation). Experiments were carried out with early generation as well as late generation tumors. Tumors were initiated in the *quadriceps femoris* of mice by inoculating aliquots of 5 × 10^5^ cells derived from intramuscular tumors initiated from the frozen stocks, and they were included in experiments when having grown to a volume of 400–600 mm^3^. Animal care and experimental procedures were approved by the Institutional Committee on Research Animal Care and were conducted according to the Interdisciplinary Principles and Guidelines for the Use of Animals in Research, Marketing, and Education (New York Academy of Sciences, New York, NY, USA).

### Tumor growth and lymph node metastasis

Tumor volume (*V*) and tumor volume doubling time (*T*_d_) were calculated as *V* = π/6 × *a* × *b* × *c* and *T*_d_ = ln2 × *t*/(ln*V*_t_ – ln*V*_0_), where *a*, *b*, and *c* represent three perpendicular tumor diameters measured with calipers, and *V*_t_ and *V*_0_ represent tumor volume at time *t* and time 0, respectively. Euthanized mice were examined for lymph node metastases in six pairs of lymph nodes (*i.e.*, popliteal lymph nodes, inguinal lymph nodes, proper axillary lymph nodes, accessory axillary lymph nodes, medial iliac lymph nodes, and renal lymph nodes), as described elsewhere [46]. The presence of metastatic growth in lymph nodes was confirmed by histological examination.

### Immunohistochemical detection of tumor hypoxia, microvessels, and collagen-I

Histological sections were prepared by standard procedures and stained with hematoxylin and eosin or immunostained for hypoxic tissue, blood vessels, or collagen-I. Pimonidazole [1-[(2-hydroxy-3-piperidinyl)-propyl]-2-nitroimidazole], injected as described earlier [47], was used as a marker of tumor hypoxia, and CD31 was used as a marker of blood vessel endothelial cells. An anti-pimonidazole rabbit polyclonal antibody (Professor James A. Raleigh, University of North Carolina, Chapel Hill, NC, USA), an anti-mouse CD31 rabbit polyclonal antibody (Abcam, Cambridge, UK), or an anti-collagen-I rabbit polyclonal antibody (Abcam) was used as primary antibody. Quantitative studies were carried out on preparations cut through the central regions of tumors, and three sections of each staining were analyzed for each tumor. Microvessels were scored as described by Weidner [28]. Fraction of pimonidazole-positive tissue and fraction of collagen-I-positive tissue were assessed by image analysis [48] and were defined as the area fractions of the non-necrotic tissue showing positive staining.

### Magnetic resonance imaging

MRI was carried out by using a Bruker Biospec 7.05-T bore magnet and a mouse quadrature volume coil (Bruker Biospin, Ettlingen, Germany). The tumors were positioned in the isocenter of the magnet and were imaged with axial slices covering the entire volume. The mice were given gas anesthesia (~4.0% Sevofluran in O_2_; Baxter, IL, USA) at a flow rate of 0.5 l/min during imaging. Respiration rate and body core temperature were monitored continuously by using an abdominal pressure sensitive probe and a rectal temperature probe (Small Animal Instruments, New York, NY, USA). The body core temperature was kept at 37° C by automated hot air flow regulation, and the gas anesthesia was adjusted manually to maintain a stable respiration rate.

DW-MRI was carried out as described previously [49]. Briefly, we applied a diffusion-weighted single-shot fast spin echo pulse sequence (RARE) with a repetition time (TR) of 1300 ms, an echo time (TE) of 26 ms, an image matrix of 64 × 64, a field of view (FOV) of 3 × 3 cm^2^, a slice thickness of 0.7 mm, and a slice gap of 0.3 mm. Four diffusion-weightings with diffusion encoding constants (*b*) of 200, 400, 700, and 1000 s/mm^2^, a diffusion gradient duration of 7 ms, and a diffusion separation time of 14 ms were used. Values of *b* ranging from 200 to 1000 s/mm^2^ were chosen to avoid perfusion effects [50, 51]. Diffusion sensitization gradients were applied in three orthogonal directions, and ADC values were calculated for each direction by using in-house-made software developed in Matlab (MathWorks, Natick, MA, USA). Furthermore, the directional diffusion images were averaged on a voxel-by-voxel basis to non-directional diffusion images, and these non-directional images were used to calculate ADC maps.

DCE-MRI with Gd-DOTA (Dotarem, Guerbet, Paris, France) as contrast agent was performed as described earlier [49]. Briefly, a fast spin echo pulse sequence (RARE) with TRs of 200, 400, 800, 1500, 3000, and 5000 ms, a TE of 8.5 ms, an image matrix of 128 × 128, a FOV of 3 × 3 cm^2^, a slice thickness of 0.7 mm, and a slice gap of 0.3 mm was used to measure precontrast *T*_1_-values (*T*_10_-map). Gd-DOTA was diluted to a final concentration of 0.06 M and administered in the tail vein in a bolus dose of 5.0 ml/kg body weight during a period of 5 s by using an automated infusion pump (Harvard Apparatus, Holliston, MA, USA). A three-dimensional SPGR pulse sequence (3D-FLASH) with a TR of 10 ms, a TE of 2.07 ms, a flip angle (α) of 20°, an image matrix of 128 × 128 × 10, and a FOV of 3 × 3 × 1 cm^3^ was used to produce postcontrast *T*_1_-weighted images at a temporal resolution of 14.8 s. Numerical values of *K*^trans^ were determined on a voxel-by-voxel basis by using the Tofts pharmacokinetic model [52]. Calculation of Gd-DOTA concentrations and pharmacokinetic modeling were done with in-house-made software developed in Matlab (MathWorks).

### Quantitative PCR

Total RNA was isolated from tumor tissue stabilized in RNA*later* RNA Stabilization Reagent (Qiagen, Hilden, Germany). RNA isolation and cDNA synthesis were performed as described previously [53]. The RT^2^ Profiler PCR Arrays Human Angiogenesis (PAHS-024Z) and Human Cancer Stem Cells (PAHS-176Z) from SABiosciences (Frederick, MD, USA) were used for expression profiling of angiogenesis-related and cancer stem cell-related genes, respectively. Real-time PCR was performed as described earlier [53]. Fold difference in gene expression was calculated by using the ΔΔC_T_-method [54]. A C_T_-value of 35 (15 cycles above the positive PCR control) was set as detection limit. Each C_T_-value of a tumor was normalized to the mean C_T_-value of the housekeeping genes (ΔC_T_ = C_T_^gene of interest^ - C_T_^mean of housekeeping genes^). The normalized gene expression level of each PDX model was calculated from three tumors as 2 ^-mean ΔCT^.

### Statistical analysis

Data are shown as mean ± standard error. Comparisons of data were carried out by using the Student *t* test (single comparisons) or by one-way ANOVA followed by the Bonferroni's test (multiple comparisons) when the data complied with the conditions of normality and equal variance. Under other conditions, comparisons were carried out by nonparametric analysis using the Mann–Whitney rank-sum test (single comparisons) or by Kruskal–Wallis ANOVA on ranks followed by the Dunn's test (multiple comparisons). The Kolmogorov-Smirnov method and the Levene's method were used to test for normality and equal variance, respectively. Probability values of *P* < 0.05 were considered significant. The statistical analysis was carried out with the SigmaStat statistical software.

## References

[1] Kopetz S, Lemos R, Powis G (2012). The promise of patient-derived xenografts: the best laid plans of mice and men. Clin Cancer Res.

[2] Willey CD, Gilbert AN, Anderson JC, Gillespie GY (2015). Patient-derived xenografts as a model system for radiation research. Semin Radiat Oncol.

[3] Gould SE, Junttila MR, de Sauvage FJ (2015). Translational value of mouse models in oncology drug development. Nat Med.

[4] Hidalgo M, Amant F, Biankin AV, Budinská E, Byrne AT, Caldas C, Clarke RB, de Jong S, Jonkers J, Mælandsmo GM, Roman-Roman S, Seoane J, Trusolino L, Villanueva A (2014). Patient-derived xenograft models: an emerging platform for translational cancer research. Cancer Discov.

[5] DeRose YS, Wang G, Lin YC, Bernard PS, Buys SS, Ebbert MT, Factor R, Matsen C, Milash BA, Nelson E, Neumayer L, Randall RL, Stijleman IJ (2011). Tumor grafts derived from women with breast cancer authentically reflect tumor pathology, growth, metastasis and disease outcomes. Nat Med.

[6] Fichtner I, Rolff J, Soong R, Hoffmann J, Hammer S, Sommer A, Becker M, Merk J (2008). Establishment of patient-derived non-small cell lung cancer xenografts as models for the identification of predictive biomarkers. Clin Cancer Res.

[7] Aparicio S, Hidalgo M, Kung AL (2015). Examining the utility of patient-derived xenograft mouse models. Nat Rev Cancer.

[8] Cassidy JW, Caldas C, Bruna A (2015). Maintaining tumor heterogeneity in patient-derived xenografts. Cancer Res.

[9] Keysar SB, Astling DP, Anderson RT, Vogler BW, Bowles DW, Morton JJ, Paylor JJ, Glogowska MJ, Le PN, Eagles-Soukup JR, Kako SL, Takimoto SM, Sehrt DB (2013). A patient tumor transplant model of squamous cell cancer identifies PI3K inhibitors as candidate therapeutics in defined molecular bins. Mol Oncol.

[10] Rubio-Viqueira B, Jimeno A, Cusatis G, Zhang X, Iacobuzio-Donahue C, Karikari C, Shi C, Danenberg K, Danenberg PV, Kuramochi H, Tanaka K, Singh S, Salimi-Moosavi H (2006). An *in vivo* platform for translational drug development in pancreatic cancer. Clin Cancer Res.

[11] Rygaard J, Povlsen CO (1969). Heterotransplantation of a human malignant tumor to ‘nude’ mice. Acta Pathol Microbiol Scand.

[12] Rygaard J, Povlsen CO (1974).

[13] Hoffman RM (2017). Patient-Derived Mouse Models of Cancer.

[14] Rofstad EK, Simonsen TG, Huang R, Andersen LMK, Galappathi K, Ellingsen C, Wegner CS, Hauge A, Gaustad JV (2016). Patient-derived xenograft models of squamous cell carcinoma of the uterine cervix. Cancer Lett.

[15] Rofstad EK, Huang R, Galappathi K, Andersen LM, Wegner CS, Hauge A, Gaustad JV, Simonsen TG (2016). Functional intratumoral lymphatics in patient-derived xenograft models of squamous cell carcinoma of the uterine cervix: implications for lymph node metastasis. Oncotarget.

[16] Ellingsen C, Hompland T, Galappathi K, Mathiesen B, Rofstad EK (2014). DCE-MRI of the hypoxic fraction, radioresponsiveness, and metastatic propensity of cervical carcinoma xenografts. Radiother Oncol.

[17] Hompland T, Ellingsen C, Galappathi K, Rofstad EK (2014). Connective tissue of cervical carcinoma xenografts: associations with tumor hypoxia and interstitial fluid pressure and its assessment by DCE-MRI and DW-MRI. Acta Oncol.

[18] Hiroshima Y, Zhang Y, Zhang N, Maawy A, Mii S, Yamamoto M, Uehara F, Miwa S, Yano S, Murakami T, Momiyama M, Chishima T, Tanaka K (2015). Establishment of a patient-derived orthotopic Xenograft (PDOX) model of HER-2-positive cervical cancer expressing the clinical metastatic pattern. PLoS One.

[19] Chaudary N, Pintilie M, Schwock J, Dhani N, Clarke B, Milosevic M, Fyles A, Hill RP (2012). Characterization of the tumor-microenvironment in patient-derived cervix xenografts (OCICx). Cancers (Basel).

[20] Dobbin ZC, Katre AA, Steg AD, Erickson BK, Shah MM, Alvarez RD, Conner MG, Schneider D, Chen D, Landen CN (2014). Using heterogeneity of the patient-derived xenograft model to identify the chemoresistant population in ovarian cancer. Oncotarget.

[21] Hoffman RM (2015). Patient-derived orthotopic xenografts: better mimic of metastasis than subcutaneous xenografts. Nat Rev Cancer.

[22] Pearson AT, Finkel KA, Warner KA, Nör F, Tice D, Martins MD, Jackson TL, Nör JE (2016). Patient-derived xenograft (PDX) tumors increase growth rate with time. Oncotarget.

[23] Hanahan D, Weinberg RA (2011). Hallmarks of cancer: the next generation. Cell.

[24] Barkan D, Green JE, Chambers AF (2010). Extracellular matrix: a gatekeeper in the transition from dormancy to metastatic growth. Eur J Cancer.

[25] Ramaswamy S, Ross KN, Lander ES, Golub TR (2003). A molecular signature of metastasis in primary solid tumors. Nat Genet.

[26] Vaupel P, Mayer A (2007). Hypoxia in cancer: significance and impact on clinical outcome. Cancer Metastasis Rev.

[27] Bao B, Azmi AS, Ali S, Ahmad A, Li Y, Banerjee S, Kong D, Sarkar FH (2012). The biological kinship of hypoxia with CSC and EMT and their relationship with deregulated expression of miRNAs and tumor aggressiveness. Biochim Biophys Acta.

[28] Weidner N (1995). Intratumor microvessel density as prognostic factor in cancer. Am J Pathol.

[29] Vermeulen PB, Gasparini G, Fox SB, Colpaert C, Marson LP, Gion M, Beliën JA, de Waal RM, Van Marck E, Magnani E, Weidner N, Harris AL, Dirix LY (2002). Second international consensus on the methodology and criteria of evaluation of angiogenesis quantification in solid human tumours. Eur J Cancer.

[30] De Palma M, Biziato D, Petrova TV (2017). Microenvironmental regulation of tumour angiogenesis. Nat Rev Cancer.

[31] Kim HS, Kwon HJ, Jung I, Yun MR, Ahn MJ, Kang BW, Sun JM, Kim SB, Yoon DH, Park KU, Lee SH, Koh YW, Kim SH (2015). Phase II clinical and exploratory biomarker study of dacomitinib in patients with recurrent and/or metastatic squamous cell carcinoma of head and neck. Clin Cancer Res.

[32] Wu SH, Lu S, Tao HJ, Zhang L, Lin WF, Shang HX, Xie J (2011). Correlation of polymorphism of IL-8 and MMP-7 with occurrence and lymph node metastasis of early stage cervical cancer. J Huazhong Univ Sci Technolog Med Sci.

[33] Wu S, Shang H, Cui L, Zhang Z, Zhang Y, Li Y, Wu J, Li RK, Xie J (2013). Targeted blockade of interleukin-8 abrogates its promotion of cervical cancer growth and metastasis. Mol Cell Biochem.

[34] Ji H, Cao R, Yang Y, Zhang Y, Iwamoto H, Lim S, Nakamura M, Andersson P, Wang J, Sun Y, Dissing S, He X, Yang X, Cao Y (2014). TNFR1 mediates TNF-α-induced tumour lymphangiogenesis and metastasis by modulating VEGF-C/VEGFR3 signalling. Nat Commun.

[35] Keeley EC, Mehrad B, Strieter RM (2010). CXC chemokines in cancer angiogenesis and metastases. Adv Cancer Res.

[36] Huang R, Rofstad EK (2017). Cancer stem cells (CSCs), cervical CSCs and targeted therapies. Oncotarget.

[37] Sampieri K, Fodde R (2012). Cancer stem cells and metastasis. Semin Cancer Biol.

[38] Li S, Li Q (2015). Cancer stem cells, lymphangiogenesis, and lymphatic metastasis. Cancer Lett.

[39] Chen Z, Li S, Huang K, Zhang Q, Wang J, Li X, Hu T, Wang S, Yang R, Jia Y, Sun H, Tang F, Zhou H (2013). The nuclear protein expression levels of SNAI1 and ZEB1 are involved in the progression and lymph node metastasis of cervical cancer via the epithelial-mesenchymal transition pathway. Hum Pathol.

[40] Yang MH, Wu MZ, Chiou SH, Chen PM, Chang SY, Liu CJ, Teng SC, Wu KJ (2008). Direct regulation of TWIST by HIF-1 alpha promotes metastasis. Nat Cell Biol.

[41] Chen HF, Huang CH, Liu CJ, Hung JJ, Hsu CC, Teng SC, Wu KJ (2014). Twist1 induces endothelial differentiation of tumour cells through the Jagged1-KLF4 axis. Nat Commun.

[42] Chen HF, Wu KJ (2016). Endothelial transdifferentiation of tumor cells triggered by the Twist1-Jagged1-KLF4 axis: relationship between cancer stemness and angiogenesis. Stem Cells Int.

[43] Hale AT, Tian H, Anih E, Recio FO, Shatat MA, Johnson T, Liao X, Ramirez-Bergeron DL, Proweller A, Ishikawa M, Hamik A (2014). Endothelial Kruppel-like factor 4 regulates angiogenesis and the Notch signaling pathway. J Biol Chem.

[44] Rofstad EK, Rasmussen H, Galappathi K, Mathiesen B, Nilsen K, Graff BA (2002). Hypoxia promotes lymph node metastasis in human melanoma xenografts by up-regulating the urokinase-type plasminogen activator receptor. Cancer Res.

[45] Samouelian V, Revillion F, Alloy N, Lhotellier V, Leblanc E, Peyrat JP (2008). Measurement of mRNA of 11 biomarkers by RT-PCR to detect lymph node involvement in cervical cancer. Int J Biol Markers.

[46] Andersen LM, Wegner CS, Simonsen TG, Huang R, Gaustad JV, Hauge A, Galappathi K, Rofstad EK (2017). Lymph node metastasis and the physicochemical micro-environment of pancreatic ductal adenocarcinoma xenografts. Oncotarget.

[47] Rofstad EK, Mathiesen B, Henriksen K, Kindem K, Galappathi K (2005). The tumor bed effect: increased metastatic dissemination from hypoxia-induced up-regulation of metastasis-promoting gene products. Cancer Res.

[48] Rofstad EK, Galappathi K, Mathiesen B, Ruud EB (2007). Fluctuating and diffusion-limited hypoxia in hypoxia-induced metastasis. Clin Cancer Res.

[49] Gaustad JV, Simonsen TG, Smistad R, Wegner CS, Andersen LMK, Rofstad EK (2015). Early effects of low dose bevacizumab treatment assessed by magnetic resonance imaging. BMC Cancer.

[50] Gaustad JV, Pozdniakova V, Hompland T, Simonsen TG, Rofstad EK (2013). Magnetic resonance imaging identifies early effects of sunitinib treatment in human melanoma xenografts. J Exp Clin Cancer Res.

[51] Padhani AR, Liu G, Koh DM, Chenevert TL, Thoeny HC, Takahara T, Dzik-Jurasz A, Ross BD, Van Cauteren M, Collins D, Hammoud DA, Rustin GJ, Taouli B, Choyke PL (2009). Diffusion-weighted magnetic resonance imaging as a cancer biomarker: consensus and recommendations. Neoplasia.

[52] Tofts PS, Brix G, Buckley DL, Evelhoch JL, Henderson E, Knopp MV, Larsson HB, Lee TY, Mayr NA, Parker GJ, Port RE, Taylor J, Weisskoff RM (1999). Estimating kinetic parameters from dynamic contrast-enhanced T1-weighted MRI of a diffusable tracer: standardized quantities and symbols. J Magn Reson Imaging.

[53] Simonsen TG, Gaustad JV, Leinaas MN, Rofstad EK (2012). High interstitial fluid pressure is associated with tumor-line specific vascular abnormalities in human melanoma xenografts. PLoS One.

[54] VanGuilder HD, Vrana KE, Freeman WM (2008). Twenty-five years of quantitative PCR for gene expression analysis. Biotechniques.

